# Three Cases of Arteritic Anterior Optic Neuropathy Several Months after COVID-19 Vaccination

**DOI:** 10.1155/2023/8845850

**Published:** 2023-09-11

**Authors:** Yuichi Yamamoto, Ken Ogino, Satoshi Yasuhara, Yu Kawashima, Toshiya Miki

**Affiliations:** Department of Ophthalmology, Japanese Red Cross Wakayama Medical Centre, Wakayama, Japan

## Abstract

**Background:**

Vaccines have been approved worldwide to control the coronavirus disease-19 (COVID-19). However, the postvaccination side effects remain controversial. Here, we describe three Japanese cases of arteritic anterior ischaemic optic neuropathy (AAION) following COVID-19 vaccination. *Case presentation*. The first case involved an 87-year-old woman who presented with vision loss in the right eye 2 months after her second COVID-19 vaccine and in the left eye 2 days later. The second case involved an 88-year-old woman who presented with vision loss in both eyes 3 months after receiving a second vaccine. The third case involved an 80-year-old man who presented with vision loss in the right eye 5 months after receiving a second vaccine. The C-reactive protein level and erythrocyte sedimentation rate were elevated in all patients. Biopsy of the temporal artery or auricular cartilage showed arteritic occlusion in case 2 and polychondritis in case 3. These patients were referred to a local Japanese hospital in 2021 over a period of no longer than 3 months.

**Conclusion:**

We observed three cases of AAION after the affected individuals received their second COVID-19 vaccine. Further long-term investigations of ophthalmological events after COVID-19 vaccination are warranted.

## 1. Introduction

Arteritic anterior ischaemic optic neuropathy (AAION) is well known to be related to giant cell arteritis (GCA) or other autoimmune diseases and can lead to optic nerve ischaemia and blindness [1]. AAION accounts for 5–10% of all anterior ischaemic optic neuropathies but is very rare in Japan [2, 3]. Many vaccines have been approved worldwide to control the coronavirus disease-19 (COVID-19) pandemic. In Japan, the Pfizer and Moderna vaccines are mainly used, and vaccination is recommended. Vaccines have resulted in lower mortality or morbidity due to COVID-19; however, there have been many reports of autoimmune and other diseases, such as arteritis, occurring after vaccination [4–10], and there is still debate regarding the side effects of vaccination. In this report, we describe three cases of AAION that were possibly associated with the second dose of the COVID-19 vaccine.

## 2. Case Presentation

### 2.1. Case 1

**Figure 1:**
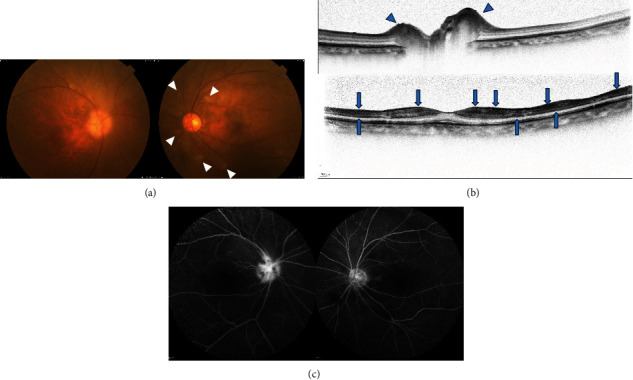
Colour photography, optical coherence tomography, and fluorescein angiography of case 1 at the first visit. (a) Margin of optic nerve in the right eye is enlarged and blurred. There are cotton-wool spots around the disc in the left eye (white arrowheads). Superior temporal macula area in the left eye shows a cherry red spot due to whitening of the retina. (b) Horizontal optical coherence tomography scan of the optic nerve in the right eye (above) shows thickening of the nerve fibre layer (blue arrowheads). Vertical scan of the macula in the left eye (below) shows high reflectivity of the ganglion cell layer and inner nuclear layer (blue arrows). (c) Optic nerve hyperfluorescence in the right eye (8′58^″^) and retinal artery filling transition in the left eye (8′32^″^) by fluorescein angiography. Arm to retina time in the left eye time (38^″^).

The patient was an 87-year-old woman who experienced a sudden loss of vision in her right eye. Two days later, the patient experienced a similar loss of vision in her left eye. Therefore, she visited another hospital and was referred to the Japanese Red Cross Wakayama Medical Centre in September 2021. One month before the onset of ocular symptoms, the patient had temporal headache and jaw claudication, and jaw claudication remained at the time of the first visit. The second dose of the COVID-19 mRNA vaccine (COMIRNATY intramuscular injection, Pfizer) was administered one month before the appearance of jaw claudication and headache. At the first visit, her best-corrected visual acuity (BCVA) was no light perception in the right eye and 0.04 (logMAR 1.39) in the left eye. Slit-lamp biomicroscopy revealed no abnormalities in the anterior segment or an intermediate translucency in either eye. Fundus examination revealed optic nerve swelling in the right eye and central retinal artery occlusion in the left eye (Figure 1(a)). Optical coherence tomography (OCT) revealed optic nerve swelling in the right eye and inner retinal hyperreflectivity and oedema in the left eye (Figure 1(b)). Fluorescein angiography (FA) revealed hyperfluorescence of the optic nerve in the right eye and filling transition of the retinal arteries in the left eye (Figure 1(c)). Contrast-enhanced magnetic resonance imaging (MRI) of the brain and orbit revealed no abnormalities that could be the cause of the symptoms. Blood tests showed an elevated WBC count (10900/*μ*L), C-reactive protein (CRP) level (5.32 mg/dL, and erythrocyte sedimentation rate (ESR) (25 mm/30 min, 60 mm/1 h, and 73 mm/2 h). Antinuclear antibody, SS-A/B antibody, myeloperoxidase anti-neutrophil cytoplasmic antibody (MPO-ANCA), and anti-aquaporin4 antibody test results were negative, and the anti-cardiolipin antibody test results showed a slight but nonsignificant elevation. Temporal artery biopsy was not performed because it was considered invasive. Colour Doppler ultrasonography revealed right temporal artery stenosis. Based on these findings, the patient was diagnosed with AAION. She was hospitalised and administered steroid pulse therapy (methylprednisolone, 1000 mg for 3 days). After steroid pulse therapy, she was started on 30 mg of oral prednisolone, with a tapering dose of 5 mg per 2 weeks. Her blood tests one month later showed a decrease in her CRP level (0.64 mg/dL) and ESR (7 mm/30 min, 16 mm/1 h, 34 mm/2 h). Unfortunately, this intensive therapy did not result in the recovery of visual acuity, but she continued with 5 mg of prednisolone without a relapse of AAION for 1 year.

### 2.2. Case 2

**Figure 2:**
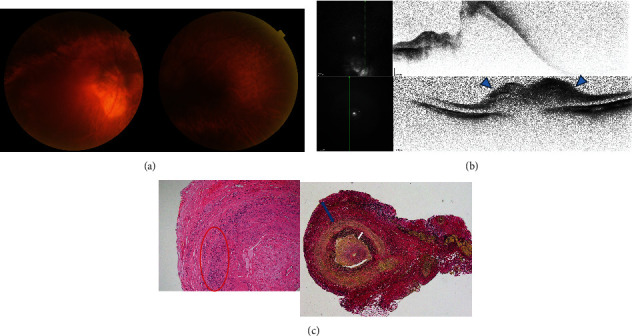
Colour photography, optical coherence tomography, and temporal artery biopsy of case 2. (a) The tilted disc in the high myopic right eye seems to be difficult in evaluation with or without swelling. Dense nuclear cataracts obscure disc swelling in the left eye. (b) Vertical scan optical coherence tomography image in the right eye (above) is unclear due to poor fixation and tilted disc. Vertical scan in the left eye (below) shows optic nerve swelling (blue arrowheads). (c) Haematoxylin and eosin stain (left) shows neutrophil/lymphocyte infiltration in the tunica media (red circle). Elastica van Gieson stain (right) shows thickening of the outer membrane (blue arrow) and separation/torn of the inner elastic plate (white arrow).

The patient was an 88-year-old woman. She became aware of sudden vision loss in both eyes, visited another hospital, and was referred to the Japanese Red Cross Wakayama Medical Center in September 2021. She had received a second dose of the COVID-19 mRNA vaccine (COMIRNATY intramuscular injection, Pfizer) 3 months previously. During the first visit, her BCVA showed no light perception or antireflection in either eye. Slit-lamp microscopy revealed dense nuclear cataracts in the anterior segment and intermediate translucency in both eyes. Fundus examination revealed a swelling of the optic nerve in the left eye (Figure 2(a)). OCT revealed optic nerve swelling in the left eye (Figure 2(b)). As the right eye had a staphyloma and a tilted disc due to high myopia, colour photography and OCT did not reveal obvious disc swelling. Contrast-enhanced MRI of the orbit showed an enhanced contrast effect on the optic nerve perimeter. However, no enhanced contrast was observed in the optic nerve. Blood tests showed elevated CRP level (2.10 mg/dL) and ESR (45 mm/30 min, 79 mm/1 h, and 89 mm/2 h). Antinuclear antibody, SS-A/B antibody, myeloperoxidase-antineutrophil cytoplasmic antibody, and anti-aquaporin4 antibody test results were negative. Temporal artery biopsy revealed neutrophil/lymphocyte infiltration and fibrinoid degeneration in the tunica media (haematoxylin and eosin staining). Elastica van Gieson staining showed that the outer membrane was thickened fibrously and the inner elastic plate was partially separated and torn (Figure 2(c)). On the basis of these findings, the patient was diagnosed with GCA and AAION. She was hospitalised and received steroid pulse therapy (methylprednisolone 1000 mg × 3 days × 3 times) and prostaglandin E1 (60 *μ*g × 7 days); however, her vision did not improve. After steroid pulse therapy, she was treated with 30 mg of orally administered prednisolone. She continued to receive 5 mg of prednisolone for more than 1 year.

### 2.3. Case3

**Figure 3:**
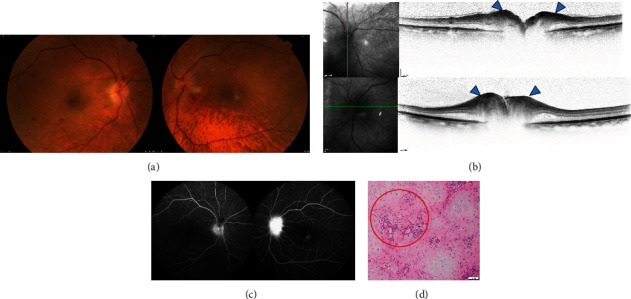
Colour photography, optical coherence tomography, and fluorescein angiography of case 3 before treatment. (a) Retinal haemorrhage predominantly around the optic disc was seen in both eyes. The inferior disc margin in the right eye is blurred. The superior temporal disc margin in the left eye is enlarged and blurred. (b) Vertical optical coherence tomography scan in the right eye and horizontal scan in the left eye show thickening of the retinal nerve fibre at disc. (c) Fluorescein angiography reveals vigorous leakage at the disc in the left eye (6′55^″^) and mild staining of the disc in the right eye (6′39^″^). Arm-to-retina time in the left eye (21^″^). (d) Auricular cartilage biopsy shows fibrosis between cartilage tissues, mild inflammatory infiltration, and haemosiderin deposition around blood vessels (red circle).

**Figure 4:**
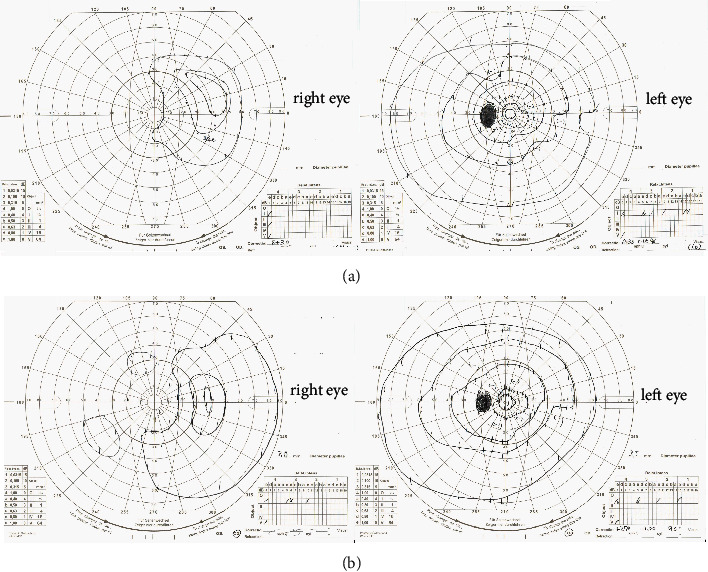
Goldmann perimeter tests before (a) and after (b) treatment. A partial recovery of the right eye is seen.

The patient was an 80-year-old man who became aware of decreased vision in his right eye and visited another hospital. Because of optic nerve swelling in each eye, he was referred to the Japanese Red Cross Wakayama Medical Center in November 2021. He had also received his second COVID-19 mRNA vaccine (COMIRNATY intramuscular injection, Pfizer) five months earlier. Four months before the onset of ocular symptoms, the patient experienced headache, stiff shoulders, swelling, and deformity of both ears, followed by pain in the right finger. At the first visit, his BCVA was 0.02 (logMAR 1.69) in the right eye and 1.2 (logMAR -0.07) in the left eye. Slit-lamp microscopy revealed no abnormalities in the anterior cortex or intermediate translucency in either eye. Fundus examination revealed optic nerve swelling and retinal haemorrhage in both eyes (Figure 3(a)). OCT revealed optic nerve swelling in both eyes (Figure 3(b)). FA revealed optic nerve hyperfluorescence in both eyes (Figure 3(c)), which was more pronounced in the left eye. Goldmann perimeter tests revealed that the temporal visual field was preserved in the right eye (Figure 4(a)). Contrast-enhanced MRI of the head revealed bilateral auricular and external auditory canal wall thickening and abnormal contrast effects. There was no enhanced contrast effect of the optic nerve on contrast-enhanced orbital MRI. Blood tests showed an elevated WBC count (9500/*μ*L), CRP level (9.8 mg/dL), and ESR (25 mm/30 min, 66 mm/1 h, 81 mm/2 h). The antinuclear antibody, SS-A/B antibody, MPO-ANCA, and anti-aquaporin4 antibody test results were negative, whereas the anti-cardiolipin antibody test results showed a slight but nonsignificant elevation. Temporal artery biopsy showed no evidence of vasculitis. Auricular cartilage biopsy showed fibrosis between the cartilage tissues, mild inflammatory infiltration, and hemosiderin deposition around the blood vessels, and a diagnosis of recurrent polychondritis was made (Figure 3(d)). Based on these findings, the patient was diagnosed with AAION complicated with recurrent polychondritis. He was hospitalised and administered steroid pulse therapy (methylprednisolone, 1000 mg for 3 days). Although his subjective symptoms improved, his vision remained unchanged. Goldmann perimeter tests revealed partial recovery from the previous visual field defect (Figure 4(b)). After steroid pulse therapy, the patient was treated with tapering therapy, starting with 55 mg of oral prednisolone and continuing at 5 mg. No relapse of AAION during tapering was observed.

## 3. Discussion

Of the three cases of AAION suspected to have been caused by COVID-19 vaccines, two were due to GCA and one was due to polychondritis. Many reports have suggested that GCA may occur after COVID-19 vaccination, and many have suspected an association with COVID-19 vaccines [10–14]. We did not find any reports of an association between polychondritis and COVID-19 vaccines. The fact that three cases of AAION, a rare disease in Japan before COVID-19 vaccination programs were implemented [2], were observed for three months might show an association with COVID-19 vaccines.

In the first case, the patient developed jaw claudication and headache one month after receiving her second vaccine, and then, one month later, she experienced a sudden loss of vision in her right eye, followed by a loss of vision in the left eye. The disease course is common in GCA. GCA can explain the retinal artery occlusion in the left eye.

The second case was diagnosed using temporal artery biopsy as giant cell arteritis with OCT findings of the bilateral optic nerves and AAION in both eyes. Jaw claudication and headache were not observed.

In the third case, the patient developed AAION in both eyes five months after receiving the second COVID-19 vaccine. FA findings showed optic nerve hyperfluorescence in both eyes; however, visual acuity was not lost in the left eye, and the patient maintained good vision. The patient also had progressive auricular deformities, and MRI and an auricular cartilage biopsy indicated polychondritis. Polychondritis is associated with AAION [15]. Although no association with COVID-19 vaccines was noted, the possibility of an association with COVID-19 vaccines is high because polychondritis is a rare autoimmune disease.

Although the exact frequency of AAION in Japan is unknown, data from the Japan Intractable Disease Information Centre indicate that approximately 1700 people have been registered with giant cell arteritis, which is the main cause of AAION [16], which means that approximately 13.6 people per million in the Japanese population had GCA in 2020. The frequency of temporal arteritis complicated by AAION is reported to be 21.2% [17], and its prevalence is estimated to be approximately 2.89 per million people. The total population of Wakayama Prefecture, where our centre is located, is approximately 920,000, and three new cases of AAION in six months are clearly a high frequency. The three cases were reported to occur after COVID-19 vaccination, which indicates “possible” drug reaction based on the Naranjo scale. In addition, there have been several reports of suspected complications after COVID-19 vaccination, including autoimmune hepatitis [4], thrombocytopenia [5], cutaneous vasculitis [6], and autoimmune nephritis [7]. There have also been reports of the possibility of developing NAION [18–20] and AAION [21] after the COVID-19 vaccination. The reported ocular symptoms potentially associated with COVID-19 vaccines include uveitis [22], acute macular optic neuropathy [23], bilateral multifocal choroiditis [24], acute-onset central serous retinopathy [25], and corneal transplant rejection [26].

It has also been reported that after COVID-19 vaccination, platelets are activated, the coagulation system is markedly stimulated, and serious thromboembolic complications may be triggered [6, 7, 19], which may have been the cause of retinal artery occlusion in the first case. After COVID-19 vaccination, neutralising antibody levels reached a maximum at approximately two weeks and then declined. After the second vaccine, neutralising antibody levels decline to 1/10^th^ of their maximum in six months [27], but cellular immunity is reported to remain even after six months [28]. The three cases of AAION reported here occurred within six months after the affected individuals received their second vaccine, indicating that complications from the COVID-19 vaccine might occur regardless of the neutralising antibody level.

## 4. Conclusions

We encountered three cases of AAION possibly related to the COVID-19 vaccination. Since AAION is a potentially blinding disease, further studies on its association with COVID-19 vaccines are needed.

## Data Availability

The datasets generated and/or analysed in the current study are available at the Japanese Red Cross Wakayama Medical Center.
